# The Effect of [Glu][H_2_PO_4_] via Foliar Spraying on Cadmium and Arsenic Absorption and Translocation in Rice Plants

**DOI:** 10.3390/toxics13020133

**Published:** 2025-02-12

**Authors:** Jiawei Deng, Lin Fu, Yanan Wang, Changbo Zhang, Yun Deng, Xin Luo, Gilles Mailhot

**Affiliations:** 1 Key Laboratory of Original Agro-Environmental Pollution Prevention and Control, Agro-Environmental Protection Institute of Ministry of Agriculture and Rural Affairs (MARA), Tianjin 300191, China; d17736218037@163.com (J.D.); fl1004197187@163.com (L.F.); wangyanan3345@163.com (Y.W.); l_xin01@163.com (X.L.); 2 School of Environment and Ecology, Jiangnan University, Wuxi 214122, China; dengyun@jiangnan.edu.cn; 3 Institut de Chimie de Clermont-Ferrand, CNRS, Université Clermont Auvergne, F-63000 Clermont-Ferrand, France; gilles.mailhot@uca.fr

**Keywords:** [Glu][H_2_PO_4_], rice, foliar application, cadmium, arsenic

## Abstract

Rice is the main source of cadmium (Cd) and arsenic (As) in Chinese diet. The formulation of targeted agronomic interventions for mitigating Cd and As bioaccumulation in rice grains constitutes a critical pathway toward ensuring food safety and public health security. Foliar spraying technology with ionic liquids, effectively reduces Cd/As content in rice. In this study, an ionic liquid of amino acids ([Glu][H_2_PO_4_]) as a foliar conditioner was applied to two varieties of rice (X24 and Z35) to explore the mechanism of reducing the accumulation of Cd/As in rice. The results showed that [Glu][H_2_PO_4_] reduced Cd/As levels by up to 58.57% and 44.09%, respectively. [Glu][H_2_PO_4_] reduced the transfer factor from the root system to flag leaves, nodes, and other organs, thus reducing the Cd/As content in them. [Glu][H_2_PO_4_] promoted amino acid synthesis in seeds, increased Ca^2+^ level, increased *OsGLR3.1–3.5* expression, and decreased *OsLsi1–3* expression in flag leaves, thereby Cd/As was inhibited from being absorbed and transported by rice. The results demonstrated that the foliar application of [Glu][H_2_PO_4_] significantly mitigated the accumulation of Cd/As in rice. This study introduces a novel and effective strategy for reducing Cd/As accumulation in rice, hoping to enhance the safety and quality of rice crops.

## 1. Introduction

The contamination of cadmium (Cd) and arsenic (As) in agricultural soils has emerged as a persistent environmental challenge [1]. Notably, regions affected by the co-contamination of Cd and As frequently coincide with rice-growing areas, thereby posing a significant threat to rice production security in China. Rice, which serves as a staple food for over half of the global population [2], exhibits a pronounced tendency to absorb and accumulate Cd and As. The cultivation of rice in soils contaminated with Cd and As has been demonstrated to have deleterious effects on the growth of the crop. Indeed, the yield and quality of the rice are significantly diminished. Furthermore, these heavy metals may subsequently entry into the human body through diet, resulting in a range of serious health issues, including cardiovascular diseases, neurological disorders, and various cancers [3,4,5]. Consequently, it is urgent to devise effective strategies for inhibiting the enrichment of Cd and As in rice, with a view to ensuring food security [6,7,8].

As a measure of agronomic regulation, leaf surface control technology has garnered increasing attention in recent years. Compared with traditional soil remediation methods, this technology offers several advantages, including simplified operation, lower costs, and minimal disturbance to the soil environment [9,10,11]. By applying specific substances such as chelators, biologics, or other functional materials onto the leaf surfaces of rice plants, a physical or chemical barrier can be formed on or within the leaf surface. This method significantly mitigates the accumulation of Cd and As in the rice plant and enhances its resistance to these toxic elements [12,13]. Additionally, these substances may enhance the tolerance of rice to heavy-metal stress by regulating its physiological and metabolic processes, indirectly reducing heavy-metal absorption.

Rice primarily absorbs Cd and As through its root system, with the majority accumulating in the roots while a minor portion is transported to other parts [9]. The fibrous root system of *Oryza sativa* amplifies rhizospheric interface dynamics through extensive surface area proliferation, mediating the bidirectional acquisition of both essential micronutrients (Fe^2^⁺, Zn^2^⁺, and Si^4^⁺) and hazardous contaminants (Cd^2^⁺, Pb^2^⁺, and AsO_3_^3−^) [14]. Transporters in the inner and outer layers of the rice cortex are involved in the absorption, chelation, transport, and accumulation of Cd. Studies have demonstrated that Cd^2+^ may share transporters with Fe^2+^, Zn^2+^, Mn^2+^, and Ca^2+^. Glutamate-receptor-like (GLR) ion channels, which are widely distributed in biological cell membranes, exhibit ligand-dependent ion transport activity, altering membrane permeability to specific ions. The increase in Cd content downregulates the expression of *OsGLR3*, thereby reducing GLRs’ preference for Ca and upregulates the expression of Mn transporter *OsNramp* [15,16]. As primarily exists in soil as As (Ⅴ) and As (III), with As (III) being more toxic than other forms. Under flooded conditions, As (III) has been observed to predominate. The research has indicated that As (III) is mainly absorbed by rice through aquaporins on root cells. Silicate is also absorbed via aquaporins, leading to antagonistic effects between As (III) and silicate as they compete for aquaporin binding sites [17]. Studies have shown that NIPs (Lsi1 and Lsi2) in rice facilitate the influx of As (III) into root cells [18]. Several studies have demonstrated that the application of ionized solutions containing essential elements such as Mn and Zn to leaf surfaces can enhance the competitive uptake of these essential elements over Cd by modulating the expression of relevant genes, thereby reducing Cd absorption and inhibiting its translocation and accumulation from vegetative tissues to reproductive organs [19,20].

Furthermore, certain amino acids and their metabolites, such as citric acid, malic acid, glutamic acid (Glu), and aspartic acid (Asp), can serve as effective leaf blocking agents to mitigate the accumulation of Cd or As in rice [21,22,23]. Citric acid plays a crucial role in enhancing plant stress tolerance by maintaining protein integrity and enzyme activity. The exogenous supplementation of malic acid can facilitate the transformation process of malate-aspartic acid, promote the synthesis of glutamic acid and aspartic acid, enhance the ability of rice vegetative organs to intercept Cd, and effectively reduce Cd content in rice [24,25,26,27,28]. However, there are few research studies on the leaf surface control technology of synchronous Cd/As reduction. Therefore, it is very important to develop an environmentally friendly foliar conditioner that reduces Cd/As accumulation.

The exogenous application of Glu can improve the expression of genes related to physiological metabolism and defense response in rice, greatly reduce the absorption and transport of Cd in rice, improve the tolerance of rice to Cd, enhance the antioxidant system, and thus reduce the damage of Cd to rice crops [29]. A study has shown that Glu can reduce the adverse effects of As on rice by improving the activity of antioxidant enzymes, promoting amino acid metabolism and nitrogen assimilation [30]. Therefore, Glu has important research significance for simultaneously reducing Cd and As pollution in rice crops. However, due to the poor solubility of Glu in water, exogenous Glu cannot be effectively absorbed and utilized by rice. Amino acid ionic liquids provide a viable solution to this problem. Amino acid ionic liquids are salts composed of amino acids or their derivatives as precursors, combined with non-toxic and harmless inorganic anions [31]. The research studies have shown that amino acid ionic liquid has a good removal effect on heavy metals [32]. The [Glu][H_2_PO_4_] used in this research is a kind of amino acid ionic liquid synthesized by the one-step method of glutamate and phosphoric acid. Phosphoric acid can form precipitates with Cd and As, thereby reducing their bioavailability. [Glu][H_2_PO_4_] has the characteristics of good water solubility, strong stability, and green environmental protection and is rich in the Glu and phosphorus elements necessary for plant growth. Up until now, there has been no documentation on the application of [Glu][H_2_PO_4_] as a foliar application to mitigate Cd and As content in rice grains. Therefore, this study employs the highly water-soluble ionic liquid [Glu][H_2_PO_4_] as a leaf surface conditioner. Through pot experiments, the effects of [Glu][H_2_PO_4_] on heavy-metal Cd/As and essential elements in various rice organs were investigated, and the mechanisms underlying its Cd/As-reducing effects were analyzed, aiming to provide a theoretical basis for the development of new Cd/As-reducing foliar fertilizers.

## 2. Materials and Methods

### 2.1. The Preparation of [Glu][H_2_PO_4_]

**Scheme 1:**
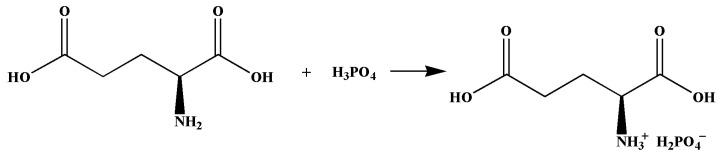
Synthetic form of [Glu][H_2_PO_4_].

The preparation of [Glu][H_2_PO_4_] was conducted in accordance with the methodologies described in references (Scheme 1) [33]. Glutamate and phosphoric acid was mixed and stirred evenly according to the molar ratio of 1:1, fully reacted for 2 h, and then the liquid was transferred to a round-bottomed flask and distilled under pressure with a rotary evaporator at room temperature to finally obtain [Glu][H_2_PO_4_].

### 2.2. Plant Materials and Pot Experiment

The pot experiment was carried out in a greenhouse controlled by artificial intelligence (coordinates: 39°5′ N, 117°9′ E) at Agro-Environmental Protection Institute of the Ministry of Agriculture and Rural Affairs. The soil used for testing was collected from the surface layer (0–20 cm) of a heavily metal-polluted farmland in Hunan Province, characterized by Cd levels of 2.32 mg·kg^−1^ and As levels of 37.29 mg·kg^−1^. According to the soil environmental quality risk control standard for soil contamination of agricultural land (GB 15618-2018) [34], the risk screening value of Cd in paddy field should not exceed 0.3 mg·kg^−1^, and the risk screening value of As should not exceed 30 mg·kg^−1^. It can be seen that the farmland in this region belongs to the Cd-As complex pollution area. The experimental materials included two indica rice varieties, X24 and Z35. Seeds were initially soaked in a 5% hydrogen peroxide (H_2_O_2_) solution for 30 min, followed by rinsing with deionized water and evenly placed in seedling trays. Germination occurred in darkness at 30 °C for two days, after which the germinated seeds were grown in vermiculite using Hoagland’s nutrient solution for 28 days. Upon reaching the two-leaf stage, the rice seedlings were transplanted into plastic pots within the greenhouse. The experiment involved six treatments, each applying foliar sprays of [Glu][H_2_PO_4_] solution at varying concentrations during rice flowering: T0 (0 mmol·L^−1^), T1 (0.2 mmol·L^−1^), T2 (0.5 mmol·L^−1^), T3 (0.8 mmol·L^−1^), T4 (1.2 mmol·L^−1^), and T5 (1.5 mmol·L^−1^). The spray treatment was performed twice, with a one-day interval between the first and second sprays, and each treatment contained three repetitions. After rice maturation, the rice plants and the soil surrounding the root systems were collected. Plant samples underwent triple washing with deionized water and were dried at 70 °C for 72 h. The harvested plant samples were divided into six parts for analysis: grain, node, spike, flag leaf, stem base, and root.

### 2.3. Determination of Cd, As, K, Ca, Mg, Fe, Mn, and Zn Content

The determination of Cd and As content was conducted in accordance with the methodologies described in references [9,35]. Accurately weighed 0.25 g of plant samples were placed into the digestion tubes, followed by the addition of 7.0 mL of Metal-Oxide-Semiconductor-Grade nitric acid (MOS HNO_3_, Fengchuan Chemical Reagent Co., Ltd., Tianjin, China) and left undisturbed for over 5 h. Digestion was performed using a DigiBlock ED54 (LabTech, Beijing, China) at 110 °C for 2.5 h. After cooling to ambient temperature, 1.0 mL of H_2_O_2_ was added, and the digestion process was extended for an additional 1.5 h. Subsequently, the temperature was raised to 170 °C to complete the deacidification step. Deionized water was subsequently added to achieve a final volume of 25.0 mL. Following filtration, the samples were analyzed. For the determination of Cd, K, Ca, Mg, Fe, Mn, and Zn concentrations, Inductively Coupled Plasma Mass Spectrometry (iCAP Q ICP-MS, Thermo Scientific, Waltham, MA, USA) was utilized. For the determination of As content, the procedure was similar to that for Cd, with the exception of maintaining a constant digestion temperature of 110 °C for 4 h without the addition of H_2_O_2_. As content was subsequently measured using an Atomic Fluorescence Spectrometer (AFS-8520, Haiguang, Beijing, China). Quality assurance and quality control were maintained through the use of standard reference materials (rice flour reference material GBW(E)100350) and blank digestion samples. The recovery rate ranged from 90% to 110%, ensuring the accuracy and reliability of the data.

Soil sample digestion methods are based on the previously reported literature [35]. A total of 0.25 g soil samples was accurately weighed into the digestion tube and digested with a mixture of 30% H_2_O_2_, hydrofluoric acid (HF), and concentrated MOS HNO_3_ at a volume ratio of 1:2:7. The final digestion solution volume was adjusted to 25 mL. The solution was then centrifuged at 3000× *g* for 5 min, and the supernatant was collected for analysis. The determination methods for Cd and As followed the procedures described earlier.

The ICP-MS used a mixed standard solution (Agilent, Part # 5183-4688, Santa Clara, CA, USA) with concentrations of Ca, Fe, Mg, and K of 1000 μg·mL^−1^ and Cd, Mn, and Zn of 10 μg·mL^−1^. The concentration range of K, Ca, and Fe in the standard solution on the machine was 0~10,000 μg·L^−1^, the concentration range of Cd, Mn, and Zn was 0~100 μg·L^−1^, and the standard curve was drawn.

During the AFS detection process, 1 g·L^−1^ arsenic standard solution (provided by Agilent) was utilized. After appropriate dilution, the concentration range of As in the standard solution was set to 0~100 μg·L^−1^, and a calibration curve was constructed accordingly.

### 2.4. Determination of Amino Acids

The amino acid content was determined according to the method described in the Chinese National Standard for Food Safety (GB5009.124-2016) [36]. First, the rice grain samples were ground into a fine powder, and then 0.25 g samples was precisely weighed into the sample tubes, followed by the addition of 15.0 mL of 6 mol·L^−1^ HCl. Next, the sample tubes were cooled in the ice bath for 5 min and then filled with nitrogen and sealed. The samples were placed in a constant temperature oven and fully digested at 110 °C for 22 h. After digestion, the tubes were removed and allowed to cool to room temperature. The digestion solution was then filtered and adjusted to a final volume of 50.0 mL. Then, 1.0 mL of the digested solution was taken and dried under a stream of nitrogen in a water bath maintained at 50 °C. This process was repeated by adding 1.0 mL of deionized water to the sample vial and drying it again with nitrogen. Finally, 2.0 mL of sodium citrate buffer (pH = 2.2) was added to the sample vial and filtered through a 0.22 μm filter membrane. The filtered digest was thoroughly mixed with 50.0 μL of 0.4 mol·L^−1^ borate buffer (pH = 10.2) for 1 min to initiate the derivatization reaction, followed by the addition of 10.0 μL of OPA reagent for 3 min. The resulting derivatives were analyzed using High-Performance Liquid Chromatography (Agilent Technologies, 1200 series, HPLC system, Agilent Technologies Inc., USA) and AdvanceBio AAA amino acid analysis column (100 mm × 4.6 mm, 2.7 μm, Agilent, USA). The mobile phase composition was acetonitrile:methanol:water = 4.5: 4.5: 1, with a flow rate of 1.0 mL·min^−1^. Quantitative analysis of amino acids in the samples was performed based on the retention time and peak area of the standard samples (17 Amino Acids Mix Solution, GBW(E)100472, BZWZ, Beijing, China).

### 2.5. Determination of Transporter Gene Expression

The RNA was extracted from fresh rice samples utilizing the Plant RNA Kit (OMEGA, R6827). The isolated RNA underwent reverse transcription to generate complementary DNA (cDNA) using HiScript II Reverse Transcriptase SuperMix for qPCR (+gDNA wiper) Kit (R223, Vazyme Biotech Co., Nanjin, China). Gene expression levels were quantitatively analyzed with the BioRad CFX Manager System (BioRad, Hercules, CA, USA) in conjunction with the ChamQ^TM^ Universal SYBR qPCR Master (Q711, Vazyme Biotech Co., Nanjin, China). Primers were designed based on pertinent literature references [37,38,39], and their specific sequences can be found in Table 1. The relative gene expression was calculated using the 2^−ΔΔCt^ method [40].

### 2.6. Statistical Analysis

Microsoft Excel 2016 were used for data analysis. The experimental results are expressed as “mean ± standard deviation (SD)”. Data visualization and statistical significance analysis were performed using R language (R 4.2.1), and univariate analysis of variance and Duncan’s multiple comparison (*p* < 0.05) were used to evaluate the significant differences and correlations between the experimental groups. Additionally, correlation analysis utilized the Pearson correlation coefficient method.

## 3. Results

### 3.1. Effects of [Glu][H_2_PO_4_] Foliar Application on Cd and As Content in Rice

**Figure 1:**
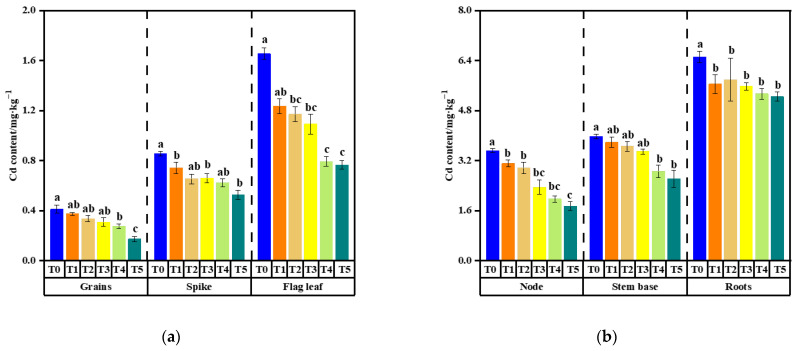

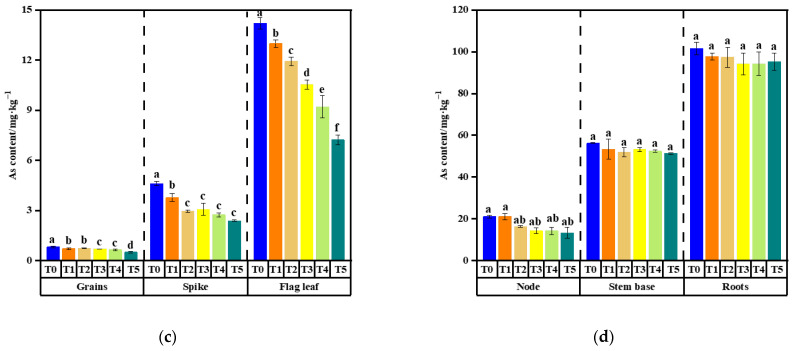
Effects of varying [Glu][H_2_PO_4_] concentrations on Cd (**a**,**b**) and As (**c**,**d**) accumulation in X24 rice organs. Treatments T0 to T5 correspond to foliar applications of 0, 0.2, 0.5, 0.8, 1.2, and 1.5 mmol·L^−1^ [Glu][H_2_PO_4_], respectively. Error bars represent standard deviations, and letters (a–f) denote significant differences among treatments (*p* < 0.05).

**Figure 2:**
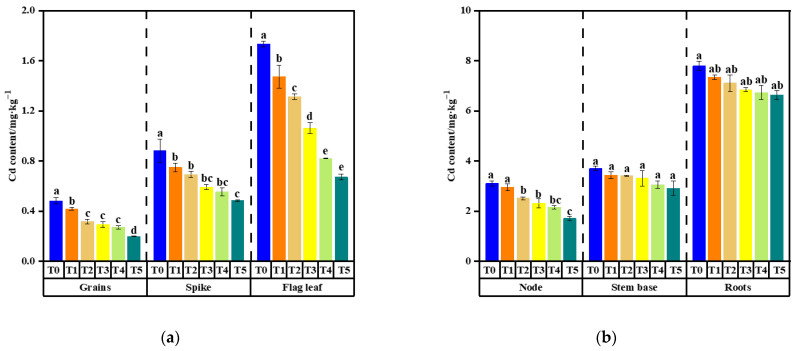

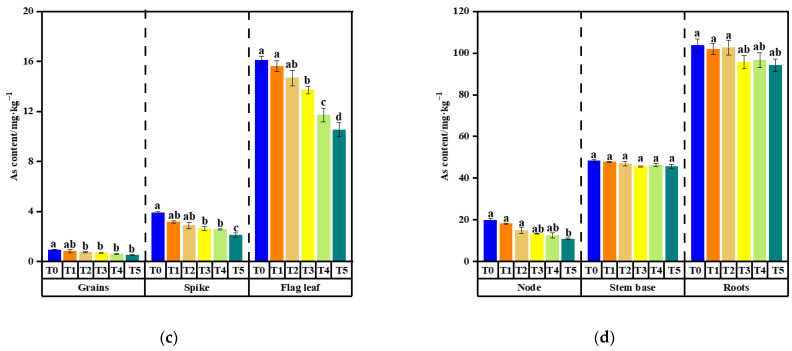
Effects of varying [Glu][H_2_PO_4_] concentrations on Cd (**a**,**b**) and As (**c**,**d**) accumulation in Z35 rice organs. Treatments T0 to T5 correspond to foliar applications of 0, 0.2, 0.5, 0.8, 1.2, and 1.5 mmol·L^−1^ [Glu][H_2_PO_4_], respectively. Error bars represent standard deviations, and letters (a–e) denote significant differences among treatments (*p* < 0.05).

The Cd and As content in rice organs at the mature stage exhibited significant variation, with aboveground tissues containing substantially lower levels of Cd/As compared to underground organs (Figure 1 and Figure 2). The roots of X24 and Z35 rice varieties displayed the highest Cd/As content, with Cd content ranging from 5.25 mg·kg^−1^ to 6.51 mg·kg^−1^ and 6.63 mg·kg^−1^ to 7.80 mg·kg^−1^, respectively. Correspondingly, As content was between 95.31 mg·kg^−1^ and 101.52 mg·kg^−1^ for X24 and 94.16 mg·kg^−1^ and 103.65 mg·kg^−1^ for Z35. The second-highest Cd/As content was observed in the stem base, while the panicle had the highest content among the aboveground tissues.

The application of [Glu][H_2_PO_4_] significantly reduced Cd and As accumulation in rice organs, with higher concentrations yielding more pronounced effects, especially in aboveground tissues. Specifically, 1.5 mmol·L^−1^ [Glu][H_2_PO_4_] showed the most effective inhibition. For X24 grains, Cd levels decreased from 0.41 to 0.17 mg·kg^−1^ (58.54% reduction), and As levels dropped from 0.82 to 0.51 mg·kg^−1^ (37.80% reduction). Similarly, in Z35 grains, Cd content declined from 0.48 to 0.20 mg·kg^−1^ (58.33% reduction), and As content decreased from 0.93 to 0.52 mg·kg^−1^ (44.09% reduction). Additionally, Cd and As levels in the flag leaves of X24 decreased by 53.33% and 49.12%, respectively, while in Z35, reductions of 61.27% and 34.68% were observed.

### 3.2. Effects of [Glu][H_2_PO_4_] Foliar Application on Cd and As Transfer Factors

The foliar application of [Glu][H_2_PO_4_] not only decreased the Cd/As content in rice organs but also significantly influenced the Cd/As transfer factors within the plant (Table 2). The order of Cd/As transport factors was TF_Stem base/Roots_ > TF_Node/Roots_ > TF_Flag leaf/Roots_ > TF_Spike/Roots_ > TF_Grains/Roots_, indicating that a substantial portion of Cd/As was intercepted by the rice stem base, thereby reducing further translocation to aerial parts. Compared with T0, the foliar spraying of [Glu][H_2_PO_4_] had a notable effect on the transfer factors of various rice organs. As the concentration of [Glu][H_2_PO_4_] increased, the Cd/As transfer factors from the roots to the aboveground parts (grain, spike, flag leaf, and node) of X24 and Z35 rice decreased significantly. Specifically, the maximum reductions in Cd transfer factors were 50.23%, 23.32%, 42.59%, and 40.62%, respectively, while the maximum reductions in As transport coefficients were 38.10%, 41.49%, 29.82%, and 39.87%, respectively. For Z35 rice, the highest decreases in Cd transfer factors from aboveground to grain parts were 46.69%, 35.46%, 54.45%, and 35.33%, respectively, and for As transfer factors, they were 36.36%, 41.49%, 29.82%, and 39.87%, respectively. These findings suggest that [Glu][H_2_PO_4_] enhances the interception of heavy metals Cd and As in rice organs and effectively inhibits their accumulation in rice grains.

### 3.3. Effects of [Glu][H_2_PO_4_] Foliar Application on Essential Elements Content in Rice

**Figure 3:**
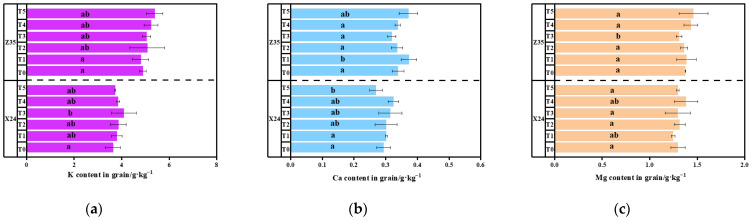

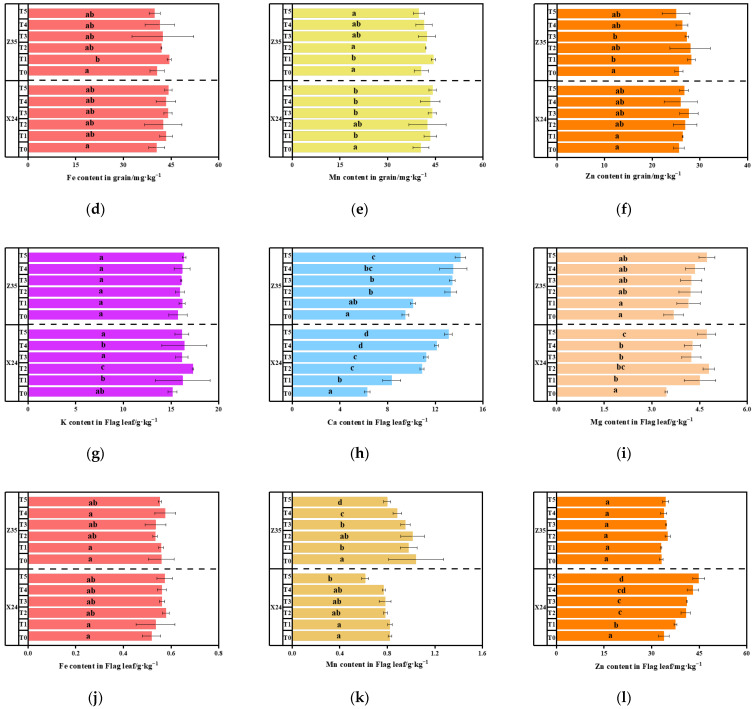
Effects of different treatments on the content of essential elements in grain (**a**–**f**) and flag leaf (**g**–**l**) of X24 and Z35 rice. The experimental groups (T0–T5) correspond to graded concentrations of 0, 0.2, 0.5, 0.8, 1.2, and 1.5 mmol·L^−1^ [Glu][H_2_PO_4_] applied through foliar treatment. Error bars denote triplicate standard deviations. Superscript letters (a–d) mark significant intergroup variations (ANOVA, *p* < 0.05).

Figure 3 delineated the nutrient profiling of six mineral elements (K, Ca, Mg, Fe, Mn, and Zn) in rice grains and flag leaves across cultivars. The results indicated that K was the most abundant element in both rice grains and flag leaves, with content ranging from 3.634 to 4.092 g·kg^−1^ in X24 grains and 4.801 to 5.390 g·kg^−1^ in Z35 grains. Mg was the second-most abundant element, followed by Ca. Notably, Ca content in grains was measured to be 0.294~0.324 g·kg^−1^ (X24) and 0.319~0.373 g·kg^−1^ (Z35), while trace elements (Fe, Mn, and Zn) maintained substantially lower content (0.02~0.05 g·kg^−1^). [Glu][H_2_PO_4_] foliar application had no significant effect on the content of these six nutrients in grain. In flag leaves, K content reached 15.153 to 17.285 g·kg^−1^ in X24 and 15.717 to 16.359 g·kg^−1^ in Z35. Ca content in X24 and Z35 flag leaves ranged from 8.299 to 13.092 g·kg^−1^ and 9.482 to 14.109 g·kg^−1^, respectively. With increasing [Glu][H_2_PO_4_] concentration, Ca content in both X24 and Z35 flag leaves increased significantly by 56.89% and 48.80%, respectively. Mn content in X24 and Z35 flag leaves was between 0.613 and 0.821 g·kg^−1^ and 0.798 to 1.041 g·kg^−1^, respectively. Contrastingly, Mn content exhibited inverse correlation with treatment intensity, declining 25.36% (X24) and 23.08% (Z35). The content of other elements did not change significantly.

**Figure 4:**
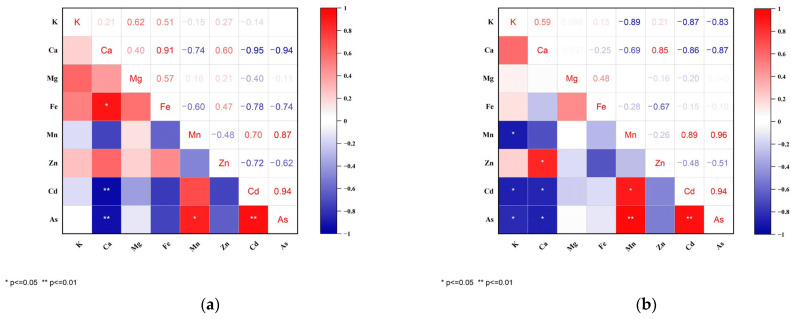
In the Pearson correlation analysis panel for the content of Cd/As and essential elements (K, Ca, Mg, Fe, Mn, and Zn) in the flag leaves of X24 (**a**) and Z35 (**b**) rice varieties under different [Glu][H_2_PO_4_] treatments. Significance thresholds: “*” means *p* < 0.05, “**” means *p* < 0.01.

Pearson correlation analysis was conducted to investigate the relationships among [Glu][H_2_PO_4_], Cd, As, and essential elements contents in flag leaves (Figure 4). The results indicated that in flag leaves of X24 rice, Cd content exhibited a strong negative correlation with Ca content (r = −0.95 **). Similarly, As content showed a significant negative correlation with Ca content (r = −0.94 **) and a significant positive correlation with manganese (Mn) content (r = 0.87 **). In the flag leaves of Z35 rice, consistent trends were observed: Cd and As contents were negatively correlated with Ca content (r = −0.86 *; r = −0.87 *), while both elements exhibited significant positive correlations with Mn content (r = 0.89 *; r = 0.96 **).

### 3.4. Effects of [Glu][H_2_PO_4_] Foliar Application on the Relative Expression Level of Transporter Genes

**Figure 5:**
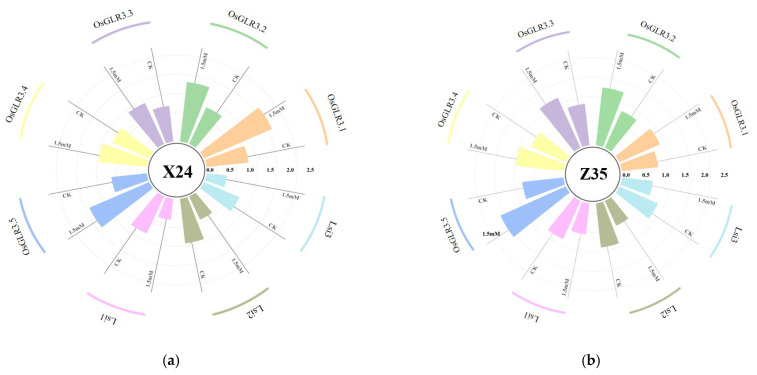
Effects of [Glu][H_2_PO_4_] on *OsGLR3.1–OsGLR3.5* and *OsLis1–3* relative expression levels in flag leaves of X24 (**a**) and Z35 (**b**) rice.

In order to delve deeper into the mechanisms governing the absorption and transport of Cd and As in rice, we carried out a quantification of the expression levels of transporter genes within the flag leaves of rice, as presented in Figure 5. The findings revealed that subsequent to the foliar application of 1.5 mmol·L^−1^ [Glu][H_2_PO_4_], the relative expression levels of *OsGLR3.1*–*OsGLR3.5* in flag leaves of X24 rice increased by 77.83%, 48.41%, 25.11%, 20.63%, and 84.8%, respectively. Consequently, the relative expression levels of *OsLsi1*–*OsLsi13* in flag leaves of X24 rice decreased by 47.80%, 47.22%, and 16.12%, respectively. Similarly, in Z35 rice flag leaves, the relative expression levels of *OsGLR3.1*–*OsGLR3.5* increased by 21.48%, 44.31%, 31.49%, 26.11%, and 73.42%, respectively, while the relative expression levels of *OsLsi1–3* decreased by 25.57%, 38.87%, and 25.74%, respectively.

### 3.5. Effects of [Glu][H_2_PO_4_] Foliar Application on the Amino Acid Content in Rice Grains

**Figure 6:**
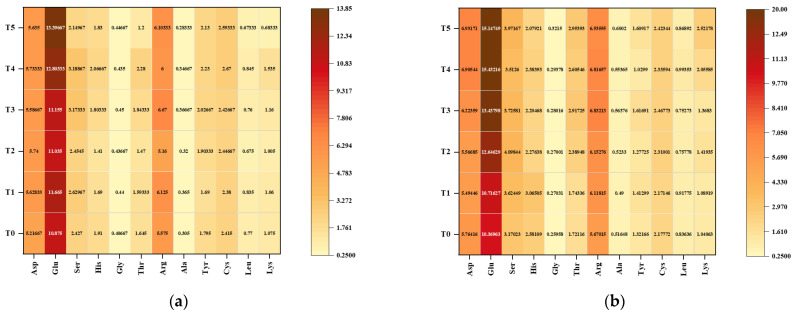
Effects of different treatments on amino acid contents in X24 (**a**) and Z35 (**b**) rice grains. These are two labeled heat maps: the horizontal coordinate represents various amino acids, and the vertical coordinate represents six treatments. The experimental groups (T0–T5) correspond to graded concentrations of 0, 0.2, 0.5, 0.8, 1.2, and 1.5 mmol·L^−1^ [Glu][H_2_PO_4_] applied through foliar treatment.

The foliar application of [Glu][H_2_PO_4_] had a remarkable impact on the content of amino acid in rice grains (Figure 6). Amino acid composition is a critical parameter for assessing the quality of rice. Glutamic acid (Glu), the most abundant non-essential amino acid in rice grains, was found to have content ranging from 10.875 g·kg^−1^ to 15.770 g·kg^−1^ in X24 and from 10.139 g·kg^−1^ to 17.147 g·kg^−1^ in Z35 rice varieties. The leaf spraying of [Glu][H_2_PO_4_] promoted the synthesis of amino acids in rice grains, with higher concentrations of [Glu][H_2_PO_4_] exhibiting a more pronounced effect. In X24 rice grains, significant increases were observed in the contents of Asp, Glu, serine (Ser), glycine (Gly), arginine (Arg), alanine (Ala), tyrosine (Tyr), and cysteine (Cys), with respective maximum increases of 9.90%, 26.90%, 31.38%, 10.65%, 19.64%, 20.22%, 33.96%, and 12.18%. In Z35 rice grains, notable increases were observed in the contents of Asp, Glu, Ser, Gly, threonine (Thr), Arg, Ala, Tyr, and Cys, with the highest increases reaching 20.31%, 46.00%, 29.27%, 23.88%, 16.60%, 20.11%, 22.45%, 25.13%, and 13.00%.

## 4. Discussion

In this study, the exogenous application of [Glu][H_2_PO_4_] can significantly reduce the content of Cd/As in rice. In contrast, Chen et al. [41] reported that spraying citric acid (CA) on the middle surface of paddy field contaminated by Cd (2.04 mg·kg^−1^) could significantly reduce the Cd content in rice, and after spraying 1 mmol·L^−1^ and 5 mmol·L^−1^ CA, the Cd content in rice grains was reduced by 37% and 52%, respectively. In this study, after spraying 1.5 mmol·L^−1^ of [Glu][H_2_PO_4_], the reduction in Cd in rice grains reached 58%, and the effect was even more significant. Similar to our findings, in the previous study, the rice field contaminated by Cd was treated by using chlorinated amino acetic acid ionic liquid ([Gly][Cl]) synthesized from glycine as a precursor, and it was found that spraying 0.8 mmol·L^−1^ [Gly][Cl] reduced the Cd content in rice grains by 39% [42]. [Glu][H_2_PO_4_] can not only reduce the content of Cd in rice to a greater extent, compared with [Gly][Cl], multiple coordination sites of Glu may enhance the chelation of Cd, and, due to the introduction of phosphoric acid, it will compete with As for the absorption pathway, reduce the intake of As [43], and realize a synchronous reduction in Cd/As.

The root system serves as the primary interface between rice plants and various rhizosphere environments, playing a critical role in nutrient uptake and heavy-metal absorption [44]. Heavy metals are predominantly absorbed by roots through the xylem, while their accumulation in rice grains primarily occurs via transport in the phloem [45]. The research has demonstrated that the root and stem base are crucial for the absorption and transport of Cd and As, with these regions exhibiting the highest concentrations of these elements [46]. The average contents of Cd and As in different parts of plant follow this order: root > stem base > node > leaf > spike > grain. The transfer of Cd/As from nutrient-rich tissues to grains is a significant source of Cd/As accumulation in rice grains. This study found that the root and stem base of rice plants contained the highest contents of Cd/As, that is, most of the toxic elements were concentrated in the underground parts. The application of [Glu][H_2_PO_4_] on the foliar reduced the transfer factors of Cd/As from the underground to the aboveground parts of rice, effectively minimizing their absorption and transport, thereby reducing their accumulation in rice grains.

The foliar application of macronutrients and micronutrients has been shown to enhance plant growth and development [47]. Due to the various absorption and transport mechanisms, the distribution of essential elements in different rice organs differs significantly. During the process of absorption and transport, antagonistic interactions occur between essential elements and Cd [48,49]. Fe can bind to As on the root surface through adsorption or co-precipitation, which can reduce the availability of As in the rhizosphere, thereby decreasing the absorption of As by rice plants. Mn influences the absorption of elements with similar ionic radii and ligand-binding properties, such as Ca, Mg, and Fe [50]. Adjusting the ratios of Ca, Mn, and Fe may be an effective strategy to mitigate Cd and As toxicity in rice. As translocation dynamics are intrinsically linked to essential nutrient transporters, particularly silicon influx carriers (OsLsi1–3) and phosphate permeases (OsPTs), where beneficial elements demonstrate 3.2–5.7-fold higher transport velocities than toxic analogs due to preferential binding affinity at conserved transporter domains [51,52]. Although essential elements exhibit competitive roles in transporters and channels compared to harmful elements, they generally have higher transport rates within the plant body. GLRs, a type of non-selective cation channel (NSCC), have a close connection with the conduction of Ca^2^⁺ and the transport of cations. [53,54]. Additionally, GLRs exhibit significantly greater selectivity for beneficial elements over harmful ones [55]. In this study, the exogenous application of [Glu][H_2_PO_4_] enhanced the expression level of *OsGLR3.1–3.5* and enhanced Ca content, which was conducive to reducing the absorption and transport of Cd in rice. Additionally, the foliar administration of [Glu][H_2_PO_4_] decreased the expression of *OsLsi1–3* genes, inhibiting As absorption and transport.

Amino acids serve as essential energy and molecular building blocks for plant and soil organism development and play a key role in biotic and abiotic stress responses during plant growth [26,56]. Studies have shown that Glu, Gly, and Cys are precursors of glutathione (GSH) and phytochelatins (PCs) [57], which can chelate Cd/As, participate in the antioxidant process in rice, and alleviate heavy-metal toxicity. For instance, Cd can form chelates with glutamate to reduce its toxicity [29]. Besides glutamic acid, aspartic acid and arginine can directly chelate Cd and As, reducing their toxicity. GSH and PCs can also chelate Cd/As through sulfur atoms on sulfhydryl groups, reducing their activity and inhibiting their transport [58]. In addition, amino acids (Glu, Gly, Cys, Asp, and Ala) can also act as signaling factors for GLRs to regulate its activity and improve its ability to recognize harmful elements [59,60]. In this study, the foliar application of [Glu][H_2_PO_4_] increased the synthesis of some amino acids such as Glu, Gly, and Cys, which was conducive to the synthesis of chelating substances such as GSH and PCs, thereby promoting the chelation of heavy-metal Cd/As and ultimately reducing its accumulation in rice grains. Concurrently, the increase in GLR activity inhibited the absorption and transport of Cd in rice.

## 5. Conclusions

The exogenous application of [Glu][H_2_PO_4_] in a certain concentration range significantly reduces the Cd and As content in rice. [Glu][H_2_PO_4_] can effectively inhibit the translocation of Cd/As between rice organs by modulating organ-specific transfer factors, thereby decreasing the accumulation of these toxic elements in rice grains. Additionally, exogenous [Glu][H_2_PO_4_] can promote the uptake of Ca^2+^ and promote the biosynthesis of (Glu, Gly, Cys, Asp, and Ala). The expression levels of *OsGLR3.1–3.5* were upregulated in rice, leading to enhanced GLR activity and increased the selectivity of GLR channels. This resulted in the inhibition of Cd absorption. Conversely, the expression of *OsLsi1–3* was downregulated, which decreased silicic acid channel activity and inhibited As entry into rice plants. In conclusion, the foliar spraying of [Glu][H_2_PO_4_] can effectively inhibit the absorption and transport of Cd/As in rice plants.

## Figures and Tables

**Table 1:** Amplification primer sequence.

Primer	Sequence (5′→3′)
*Actin1-F*	TCCATCTTGGCATCTCTCAG
*Actin1-R*	GTACCCTCATCAGGCATCTG
*OsGLR3.1-F*	AGAACCCTAAGTTCGCCGTC
*OsGLR3.1-R*	AGAGGTTGCAGGCGTGTATG
*OsGLR3.2-F*	AGCATGGCCCTAAGAGAGGA
*OsGLR3.2-R*	CTCCGACAGTGACAGGATCG
*OsGLR3.3-F*	TGGACCCAAAGATGGTGGTG
*OsGLR3.3-R*	GCGAGAACCACTCGTCATGT
*OsGLR3.4-F*	AGCAGCTTCTGGGGACTCT
*OsGLR3.4-R*	GGTATCTGAAGTACTGGCGCA
*OsGLR3.5-F*	ATTCTGCCACTGTTGACCCA
*OsGLR3.5-R*	CATGCCCAGCTGTGACTGAT
*Lsi1-F*	CGGTGGATGTGATCGGAACCA
*Lsi1-R*	CGTCGAACTTGTTGCTCGCCA
*Lsi2-F*	ATCTGGGACTTCATGGCCC
*Lsi2-R*	ACGTTTGATGCGAGGTTGG
*Lsi3-F*	CTGTATCCCTGTTGCCAGCTG
*Lsi3-R*	TAATCCGGCATGCGTACTTG

Note: F denotes the forward primer, while R indicates the reverse primer.

**Table 2:** Effects of [Glu][H_2_PO_4_] on Cd/As transfer factors in X24 and Z35 rice. The experimental groups (T0-T5) correspond to graded concentrations of 0, 0.2, 0.5, 0.8, 1.2, and 1.5 mmol·L^−1^ [Glu][H_2_PO_4_] applied through foliar treatment. Different lowercase letters (a–e) denote statistically significant variations among treatment groups (*p* < 0.05) in Cd and As transfer coefficients for both X24 and Z35 rice varieties.

Rice Variety	Element	Treatment	TF_Grains/Roots_	TF_Spike/Roots_	TF_Flag leaf/Roots_	TF_Node/Roots_	TF_Stem base/Roots_
X24	TF(Cd)	T0	0.0661 ± 0.0041 a	0.1312 ± 0.0030 a	0.2543 ± 0.0140 a	0.5606 ± 0.0237 a	0.6909 ± 0.016 a
		T1	0.0653 ± 0.0024 a	0.1317 ± 0.0141 a	0.2193 ± 0.0187 b	0.5513 ± 0.0395 a	0.6708 ± 0.0477 a
		T2	0.0583 ± 0.0046 b	0.1141 ± 0.0151 b	0.2048 ± 0.0339 b	0.5194 ± 0.0915 bc	0.6397 ± 0.0994 ab
		T3	0.0552 ± 0.0056 b	0.1182 ± 0.0040 b	0.1959 ± 0.0182 bc	0.4218 ± 0.0508 bc	0.6258 ± 0.0265 ab
		T4	0.0517 ± 0.0029 c	0.1168 ± 0.0027 c	0.1487 ± 0.0083 c	0.3699 ± 0.0291 c	0.5360 ± 0.0342 b
		T5	0.0329 ± 0.0036 d	0.1006 ± 0.0110 c	0.1460 ± 0.0033 c	0.3329 ± 0.0229 c	0.4703 ± 0.0391 b
	TF(As)	T0	0.0021 ± 0.0001 a	0.0094 ± 0.0004 a	0.0389 ± 0.0005 a	0.0958 ± 0.0029 a	0.4788 ± 0.0157 a
		T1	0.0019 ± 0.0002 a	0.0078 ± 0.0005 b	0.0383 ± 0.0020 a	0.0889 ± 0.0036 b	0.4682 ± 0.0119 a
		T2	0.0019 ± 0.0001 a	0.0070 ± 0.0004 b	0.0358 ± 0.0025 b	0.0729 ± 0.0060 c	0.4583 ± 0.0134 a
		T3	0.0019 ± 0.0001 a	0.0069 ± 0.0003 bc	0.0359 ± 0.0019 b	0.0694 ± 0.0045 c	0.4779 ± 0.0165 a
		T4	0.0017 ± 0.0002 ab	0.0066 ± 0.0003 bc	0.0295 ± 0.0004 c	0.0643 ± 0.0043 d	0.4790 ± 0.0117 a
		T5	0.0013 ± 0.0002 b	0.0055 ± 0.0007 c	0.0273 ± 0.0020 c	0.0576 ± 0.0023 e	0.4726 ± 0.0248 a
Z35	TF(Cd)	T0	0.0619 ± 0.0021 a	0.1131 ± 0.0102 a	0.2226 ± 0.0078 a	0.3982 ± 0.0133 a	0.4703 ± 0.0122 a
		T1	0.0573 ± 0.0010 ab	0.1018 ± 0.0034 ab	0.2006 ± 0.0105 ab	0.4030 ± 0.0221 a	0.4675 ± 0.0247 a
		T2	0.0446 ± 0.0044 b	0.0976 ± 0.0059 ab	0.1849 ± 0.0054 ab	0.3542 ± 0.0184 ab	0.4804 ± 0.0180 a
		T3	0.0430 ± 0.0033 b	0.0864 ± 0.0023 b	0.1553 ± 0.0053 b	0.3380 ± 0.0292 ab	0.4834 ± 0.0402 a
		T4	0.0404 ± 0.0018 bc	0.0821 ± 0.0048 b	0.1220 ± 0.0054 c	0.3211 ± 0.0217 ab	0.4545 ± 0.0379 a
		T5	0.0300 ± 0.0012 c	0.0730 ± 0.0013 c	0.1014 ± 0.0043 c	0.2574 ± 0.0048 b	0.4614 ± 0.0497 a
	TF(As)	T0	0.0022 ± 0.0001 a	0.0094 ± 0.0004 a	0.0389 ± 0.0005 a	0.0958 ± 0.0029 a	0.4718 ± 0.0157 a
		T1	0.0022 ± 0.0004 a	0.0078 ± 0.0005 b	0.0383 ± 0.0020 a	0.0889 ± 0.0036 a	0.4682 ± 0.0119 a
		T2	0.0018 ± 0.0001 ab	0.0070 ± 0.0004 b	0.0358 ± 0.0025 ab	0.0729 ± 0.0060 b	0.4583 ± 0.0134 a
		T3	0.0018 ± 0.0002 ab	0.0069 ± 0.0003 b	0.0359 ± 0.0019 ab	0.0694 ± 0.0045 b	0.4779 ± 0.0165 a
		T4	0.0016 ± 0.0001 b	0.0066 ± 0.0003 b	0.0295 ± 0.0004 b	0.0643 ± 0.0043 b	0.4790 ± 0.0117 a
		T5	0.0014 ± 0.0001 b	0.0055 ± 0.0007 c	0.0273 ± 0.0020 b	0.0576 ± 0.0023 b	0.4726 ± 0.0248 a

## Data Availability

Dataset available on request from the authors.
